# Targeting the Endoplasmic Reticulum Unfolded Protein Response to Counteract the Oxidative Stress-Induced Endothelial Dysfunction

**DOI:** 10.1155/2018/4946289

**Published:** 2018-03-14

**Authors:** Giuseppina Amodio, Ornella Moltedo, Raffaella Faraonio, Paolo Remondelli

**Affiliations:** ^1^Dipartimento di Medicina, Chirurgia e Odontoiatria “Scuola Medica Salernitana”, Università degli Studi di Salerno, 84081 Baronissi, Italy; ^2^Dipartimento di Farmacia, Università degli Studi di Salerno, 84084 Fisciano, Italy; ^3^Dipartimento di Medicina Molecolare e Biotecnologie Mediche, Università degli studi di Napoli “Federico II”, 80131 Naples, Italy

## Abstract

In endothelial cells, the tight control of the redox environment is essential for the maintenance of vascular homeostasis. The imbalance between ROS production and antioxidant response can induce endothelial dysfunction, the initial event of many cardiovascular diseases. Recent studies have revealed that the endoplasmic reticulum could be a new player in the promotion of the pro- or antioxidative pathways and that in such a modulation, the unfolded protein response (UPR) pathways play an essential role. The UPR consists of a set of conserved signalling pathways evolved to restore the proteostasis during protein misfolding within the endoplasmic reticulum. Although the first outcome of the UPR pathways is the promotion of an adaptive response, the persistent activation of UPR leads to increased oxidative stress and cell death. This molecular switch has been correlated to the onset or to the exacerbation of the endothelial dysfunction in cardiovascular diseases. In this review, we highlight the multiple chances of the UPR to induce or ameliorate oxidative disturbances and propose the UPR pathways as a new therapeutic target for the clinical management of endothelial dysfunction.

## 1. Introduction

Endothelial cells produce different vasoactive substances that control vascular homeostasis in concert with pro- and antioxidant or pro- and anti-inflammatory factors [1–3]. Among them, nitric oxide (NO) which is produced by nitric oxide synthases (NOS) and targets guanylyl cyclase of the underlying smooth muscle cells to activate the signalling of vasodilatation plays a key function in blood vessel homeostasis [4, 5]. Endothelial dysfunction (ED) occurs when vascular homeostasis is altered in favour of vasoconstriction, inflammation, and prooxidation, all factors that produce a proatherogenic and prothrombotic phenotype [3, 6]. ED is the early pathogenic event of several cardiovascular and metabolic diseases and therefore is predictive of cardiovascular events with fatal outcome [7, 8]. Reduced endothelium-dependent dilatation (EDD) is the initial signal of ED. EDD is the consequence of reduced NO bioavailability resulting from impaired NO production or increased NO degradation. In this state, endothelial NOS (eNOS) begins to generate reactive oxygen species (ROS), such as superoxide, a phenomenon known as “uncoupling” [3–5]. Furthermore, peroxynitrite (ONOO^−^) promotes nitration of the eNOS cofactor BH4 and critical antioxidants, leading to propagation of ED and endothelial cell death [9]. Similar to eNOS uncoupling, other enzymes may function as ROS sources, such as NADPH oxidase, xanthine oxidase, and the mitochondrial respiratory chain complex, giving rise to OS-induced ED, an event that occurs in several different cardiovascular diseases (CVDs) [10–14]. Increasing evidence identifies endoplasmic reticulum stress (ER stress) as another source of ROS [15, 16]. As a consequence, a growing number of studies are focused on defining the role of ER stress in OS induction aiming at understanding whether ER stress could have a role as a promoter of ED or merely worsen ED in human pathologies [14, 17–19]. In this review, we will analyse the basic mechanisms of ER production of ROS and discuss novel targets for the pharmacological therapy of CVDs derived from ED.

## 2. Endoplasmic Reticulum Function and the Control of the Redox State of the Cell

Redox homeostasis inside the cell is controlled by specialized mechanisms located in the cytosol, as well as within the peroxisomes, mitochondria, and the ER. The ER is intensely engaged in the control of folding and trafficking of secretory proteins [20]. Within the ER lumen, a quality control system (ERQC) selects properly folded from misfolded proteins that are addressed to degradation rather than to access downstream cell compartments of the secretory pathway. In this way, the ER ensures the functions of post ER compartments and controls the proteostasis and the trafficking of secretory proteins [21–24]. Under normal conditions, the ER has restricted antioxidant activity and the ER proteostasis is highly sensitive to the redox state of the cell. Several pathophysiological conditions could disturb the ER proteostasis by inducing the accumulation of misfolded or unfolded proteins within the ER [25, 26]. This condition is called ER stress and activates the signalling pathways of the unfolded protein response (UPR) [27, 28]. The UPR pathways aim to reestablish ER proteostasis throughout different outcomes: reducing ER protein load, potentiating the ER quality control, activating the ER-associated protein degradation machinery (ERAD), and, eventually, activating autophagy [29]. However, when all the adaptive responses fail, the UPR can activate the apoptotic programme [30, 31]. Since protein folding is coupled to ROS formation, the increment of folding load during ER stress strongly induces ROS production and exacerbates OS [16, 32–34]. The formation of disulfide bonds within the ER requires a stable redox environment. In order to maintain redox homeostasis during protein folding, the ER is provided with several buffering factors, such as glutathione (GSH), ascorbic acid, and flavin nucleotides. Specifically, GSH reacts with and reduces nonnative disulfide bonds, thus allowing misfolded proteins to fold again [35]. In the meantime, specific oxidoreductases such as protein disulfide isomerases (PDIs), in conjunction with the ER oxidoreductase 1 (Ero1), catalyse disulfide bond formation [36–38], but this event generates the formation of hydrogen peroxide (H_2_O_2_), the most abundant ROS produced in the ER. During ER stress, the accumulation of misfolded proteins, which requires more cycles of disulfide bond formation and isomerization, produces a higher amount of H_2_O_2_, depletes the ER GSH level, and, as a consequence, devastates the redox state of the ER [39].

## 3. The Unfolded Protein Response Pathways: Oxidative and Antioxidative Control

**Figure 1:**
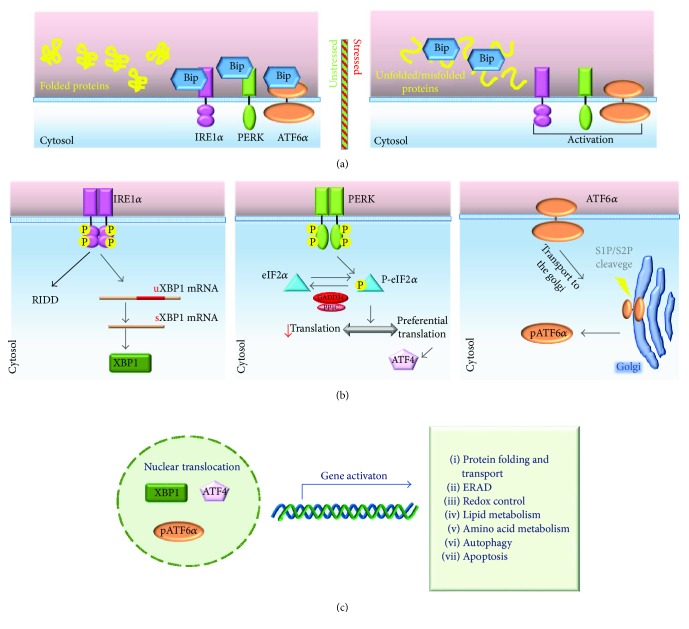
The signalling pathways of UPR. (a) During normal conditions, Bip/GRP78 binding to IRE1*α*, PERK, and ATF6*α* maintains the three transducers in an inactive state. In stressed conditions, Bip/GRP78 dissociates from IRE1*α*, PERK, and ATF6*α* to help the folding of secretory proteins and allows the activation of the transducers [28]. (b) After the release from Bip/GRP78, IRE1*α* dimerizes and autophosphorylates to activate its kinase and endoribonuclease domains [15]. Activated IRE1*α* cleaves 26 nucleotides from the mRNA encoding the X-box-binding protein 1 (XBP1) allowing the translation of XBP1 [140]. Bip/GRP78 dissociation enables also PERK activation through dimerization and *trans*-autophosphorylation. Activated PERK phosphorylates eIF2*α* at Ser51 leading to attenuation of protein synthesis, thereby reducing ER protein load. During this condition, some mRNA, such as the activating transcription factor 4 (ATF4) mRNA, are preferentially translated [141]. During severe ER stress, ATF4 strongly induces CHOP that triggers the apoptotic programme in different ways [31]. The eIF2*α*-ATF4 axis can also be activated by other cytosolic kinases allowing the regulation of global protein synthesis and the preferential translation of specific mRNA in response to different stimuli in a convergent signalling pathway known as integrated stress response (ISR) [20, 30]. ATF6*α* is the third ER stress sensor located in the ER membrane. Upon ER stress and release by Bip/GRP78, ATF6*α* is packaged into COPII vesicles and transferred to the *cis*-Golgi where it undergoes intramembrane proteolysis-specific cleavage by site 1 protease (S1P) and S2P to produce a transcriptionally active fragment (pATF6*α*). (c) XBP1, ATF4, and pATF6*α* migrate into the nucleus to activate the transcription of specific UPR genes involved in protein folding and trafficking, ERAD, cellular metabolism, autophagy, and apoptosis [20, 142]. Bip: Bip/GRP78; uXBP1: unspliced XBP1; sXBP1: spliced XBP1.

**Figure 2:**
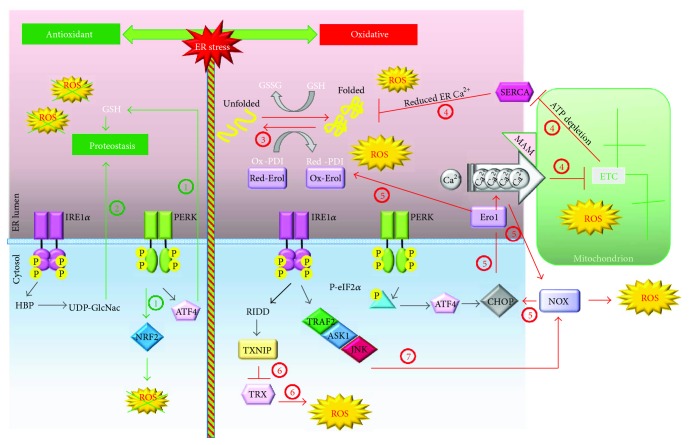
The oxidative and antioxidant programmes of UPR. The antioxidant (green lines) and oxidative (red lines) pathways of UPR are depicted on the left or on the right, respectively. The PERK and IRE1*α*/XBP1 pathways promote the maintenance of ER proteostasis as follows. (1) There is PERK-mediated activation of the antioxidant transcription factor NRF2 and the promotion of GSH synthesis [45]. (2) There is IRE1*α*/XBP1-mediated induction of the hexosamine biosynthetic pathway (HBP), which is important for the production of UDP-GlcNAc [41]. On the right, the ER stress-dependent amplification of ROS production (red lines) is depicted. (3) Following ER stress, the increased folding activity of ER augments ROS production. (4) The ER stress increases the MAM-mediated calcium flux to mitochondria that inhibits ETC and increases mitochondrial ROS production; moreover, reduced ATP synthesis from the impaired ETC affects SERCA activity and the consequent ER calcium content which in turn boosts up unfolding [143]. (5) CHOP, through the induction of Ero1, potentiates calcium efflux from the ER. The higher cytosolic calcium activates the Ca^2+^/calmodulin-dependent protein kinase II- (CaMKII-) JNK-NOX-protein kinase R (PKR) pathway, which in turn positively feedbacks on CHOP expression [47, 57]. In addition, Ero1-increased expression potentiates the oxidative protein folding and ROS production. (6) Through microRNA inhibition, the RIDD activity of IRE1 relieves the expression of TXNIP protein that blocks the antioxidant enzyme TRX [50]. (7) IRE1*α* activates the tumor necrosis factor *α*-associated receptor 2 (TRAF2)/ASK1/JNK pathway that further upregulates the NOX-dependent ROS production [48, 144]. For detailed discussion and references, see the text. Red: reduced; Ox: oxidized; TRX: thioredoxin.

The ER stress activates the UPR pathways by means of three transmembrane transducers: the inositol-requiring kinase 1 (IRE1), the pancreatic ER kinase (PERK), and the activating transcription factor 6 (ATF6) [28]. In normal conditions, the three transducers are maintained inactive by the chaperone binding immunoglobulin protein/78 kDa glucose-regulated protein (Bip/GRP78). In stressed conditions, Bip/GRP78 dissociates from IRE1, PERK, and ATF6 and allows UPR activation (Figure 1). The adaptive response induced by the UPR, if successful, can moderate ROS production within the ER, not only by simply reducing the folding demand but also by performing another compensative response consisting in the activation of genes encoding antioxidant factors (Figure 2). In particular, antioxidant control has been linked to the PERK and IRE1 pathways as shown by the work of Harding et al. [40]. They demonstrated that ATF4 is essential for GSH synthesis and, as a consequence, for the maintenance of redox balance in the ER. Moreover, the IRE1/XBP1 branch of the UPR stimulates the hexosamine biosynthetic pathway (HBP), which is essential for the production of UDP-N-acetylglucosamine (UDP-GlcNAc). This compound is crucial for the stress-induced O-GlcNAc modifications, which favour cell survival and increase the defence against ROS [41]. Besides the ATF4/GSH and the XBP1/HBP antioxidant pathways, the UPR controls the activation of a potent transcription factor involved in the antioxidant response: the nuclear factor erythroid 2-related factor 2 (NRF2) [42, 43]. Under basal conditions, NRF2 is inactivated by the Kelch-like ECH-associated protein 1 (KEAP1), which induces its degradation through the cullin3/ring box 1-depedent ubiquitin ligase complex. During OS, ROS react with specific KEAP1 cysteines inducing conformational changes that prevent the binding of de novo-produced NRF2. As a consequence, newly translated NRF2 can migrate into the nucleus to activate antioxidant gene transcription [44]. In addition to that, it is well established that OS-activated PERK could induce NRF2 phosphorylation and dissociation from KEAP1 [45] enhancing the antioxidant activity of NRF2. Since ER protein misfolding highly increases ROS, we would expect that UPR activation could preferentially reduce abnormal production of ROS. On the contrary, evidence shows that UPR pathways can even activate ROS production during ER stress and therefore aggravate the OS (Figure 2). This is the case of the PERK pathway of the UPR that activates the transcription factor C/EBP homologous protein (CHOP), which induces the expression of Ero1 that accounts for the peroxide production during the oxidative protein folding [37, 38, 46]. Additionally, CHOP expression can be enhanced by the ROS-induced activation of the NADPH oxidase (NOX) members 2 or 4, which induce the double-stranded RNA-dependent protein kinase (PKR), another activator of CHOP [47]. The PERK/CHOP axis is not the only pathway of the UPR that initiates ROS formation. In fact, the IRE1 pathway of the UPR activates the apoptosis signal-regulating kinase 1 (ASK1) [48] and ASK1 activation is also sustained by the mitochondrial ROS production deriving from c-Jun N-terminal kinase- (JNK-) mediated inhibition of the mitochondrial electron transport chain (ETC) [49]. This event leads to the persistent activation of ASK1 thus linking the activation of UPR to OS-induced apoptosis. The IRE1 pathway of the UPR also contributes to OS by increasing thioredoxin-interacting protein (TXNIP) mRNA levels throughout the reduction of the TXNIP inhibitory microRNA-17 [50], and such event makes cells more susceptible to OS, since TXNIP inhibits the antioxidant thioredoxin (TRX) enzyme. Several studies have demonstrated the fine tuning of the UPR by the OS [51, 52]. OS control of the UPR is mediated by the protein disulfide isomerases PDIA5, which reduces disulfide bonds in the luminal domain of ATF6, and PDIA6, which reduces specific cysteines of the luminal domain of PERK and IRE1. In this way, by promoting oxidation of the three UPR sensors, ROS could modulate the UPR by inhibiting the ATF6 pathway and, simultaneously, potentiating the IRE1 and PERK pathways.

## 4. The Endoplasmic Reticulum/Mitochondria Axis for Reactive Oxygen Species Production

OS activated at the ER level can be transmitted in a Ca^2+^-dependent manner to mitochondria with a consequent production of ROS. Mitochondria are connected to the ER through mitochondrial-associated ER membranes (MAMs) [53]. Across MAMs, ATP, Ca^2+^, metabolites, and ROS are rapidly transmitted from the ER to mitochondria [54]. As a consequence, the sustained calcium influx from the ER into mitochondria triggers the opening of the permeability transition pore and the release of cytochrome C. Loss of cytochrome C impairs complex III of the mitochondrial ETC with the consequent increase of ROS production [55, 56]. Moreover, Ero1 that is transcriptionally induced by CHOP during the UPR potentiates the inositol-1,4,5-trisphosphate receptor (IP3R)-mediated Ca^2+^ leakage from the ER [57, 58]. Under these circumstances, ROS production could even be enhanced by other mechanisms. Firstly, the UPR induces the expression of a truncated isoform of SERCA pumps that increase Ca^2+^ transfer to mitochondria [59]. Then, impaired ETC affects ATP production inhibiting SERCA pumps [60]. Furthermore, the ER protein sigma-1 receptor dissociates from Bip/GRP78 following calcium depletion from ER and stabilizes IP3R at MAM leading to a prolonged calcium signalling to mitochondria [61]. Next, PERK is uniquely enriched in MAMs and helps the tightening of ER-mitochondria contact sites during chronic ER stress facilitating calcium influx and ROS-mediated mitochondrial apoptosis [62, 63]. Nevertheless, ER Ca^2+^ pumps and IP3R or ryanodine receptor (RyR) channels are themselves influenced by the redox state of ER [64] together with the IP3 agonist of IP3R channels [65]. Thus, Ca^2+^-mediated mitochondrial ROS production further enhances calcium release from ER, which in turn impairs Ca^2+^-dependent chaperone activity and ER homeostasis, resulting in ER stress. Moreover, ROS themselves impair the ER oxidative protein folding. Indeed, the futile cycles of disulfide bond formation produce more ROS and, by depleting ATP, stimulate mitochondrial ROS production and so on. Taken together, these mechanisms create a vicious cycle of ER stress and mitochondrial dysfunction that boost each other and decide for apoptosis commitment.

## 5. Endoplasmic Reticulum Stress and the Unfolded Protein Response Pathways as Therapeutic Targets in the Oxidative Stress-Induced Endothelial Dysfunction

The role of the UPR pathways in the beginning of ED is a relatively recent area of investigation. Just over ten years ago Gargalovic et al. [66] were among the first to demonstrate the activation of UPR in human aortic endothelial cells exposed to oxidized phospholipids. In this work, it was demonstrated that the UPR factors ATF4 and XBP1 were both required for the activation of proinflammatory proteins and that the silencing of their expression abolished these effects. Although the authors did not demonstrate the mechanisms of the UPR induction by oxidised phospholipids, they hypothesised that an increase in OS could at least in part explain UPR activation and, in this way, they provided the first proof of the contribution of the ER stress in ED. Since then, several studies have shown the correlation of ER stress and UPR to ED in both animal and cellular models [67–70]. The failure of antioxidant therapy in decreasing cardiovascular risk in human clinical trials [71, 72] points up the importance to find new therapeutic approaches to counteract OS induced ED. Since ER stress is closely linked to OS, as discussed in depth in this review, targeting the UPR pathways or the ER stress could be a successful approach in the attempt to neutralise OS. Two possible approaches can be used to counteract OS-induced UPR. One is to modulate directly the activity of individual UPR mediators. Another consists of the activation of auxiliary pathways potentiating the adaptive response to ER stress to relieve unfolding. With reference to the last option, novel pharmacological inhibitors of ER stress-induced ED have been identified. One example is hyperhomocysteinemia. Hyperomocysteinemia is a cardiovascular risk factor associated with ED, atherosclerotic vascular diseases, and ischemic heart attacks [73]. It is well established that homocysteine (HC) induces ER stress by disrupting disulfide bond formation and that ER stress activates apoptosis in vascular cells through the upregulation of CHOP [74]. Instead, the activation of the PERK pathway of the UPR can induce endothelial detachment-mediated apoptosis through the overexpression of the T cell death-associated gene 51 (TDAG51) [75]. Recently, it has been reported that HC also impairs EDD following ER stress-mediated inhibition of the Ca^2+^-activated potassium channel [76] and that the resveratrol analogue piceatannol displays a protective effect on HC-induced ED through the NRF2-mediated upregulation of heme oxygenase 1 (HO-1) [77]. In particular, pretreatment with piceatannol significantly reduced ER stress, homocysteine-induced apoptosis, and ROS production in endothelial cells [77]. Interestingly, many natural compounds can ameliorate ED through the reduction of ER stress-induced OS. As an example, black tea extracts improved endothelial-dependent relaxation and attenuated ROS production in HC-treated rat aortae and in cultured rat aortae cells through the suppression of ER stress both in HC- and angiotensin II-induced hypertension [78]. Another compound extracted from the Chinese herb barberine showed the ability to reduce endothelial-dependent contraction in carotid arteries from spontaneous hypertensive rats through the alleviation of ER stress, the reduction of ER stress-dependent ROS production, and the downregulation of the ROS-dependent expression of cyclooxygenase-2 (COX-2) [79]. This effect depended on the activation of AMP activated protein kinase (AMPK). AMPK is a protein involved in the control of energy status, whose induction has been correlated with the mitigation of ER stress in several studies [79–82]. The upregulation of AMPK is another putative way to induce an auxiliary pathway reducing ER stress. An example of the therapeutic effect of AMPK activation is the work by Li et al. [83], in which the natural triterpenoid ilexgenin A was found to be therapeutic in high-fat diet- (HFD-) fed mice and in endothelial cells stimulated with palmitate. In these models, ilexgenin A reduced ER stress and ER stress-dependent ROS generation through the inhibition of the NOD-like receptor family pyrin domain containing 3 (NLRP3) inflammasome and this effect depended on enhanced AMPK activity. Moreover, in HFD-fed mice the oral administration of ilexgenin A improved significantly endothelial function with the recovery of EDD and NO production [83]. These results strongly suggested that AMPK activation is helpful to reduce ER stress and ED and have triggered the study of new pharmacological inducers of AMPK. Among them, aminoimidazole carboxamide riboside (AICAR), salicylate, cycloastragenol, and astragaloside-IV inhibit ER stress-dependent ROS generation and the induction of NLRP3 inflammasome in various models of palmitate-induced ED [84, 85].

Although the molecular mechanism involved in AMPK-dependent mitigation of ER stress was not fully addressed, it could be possible that the key target of the AMPK action is the inhibition of the OS-generated upstream or downstream of the ER stress, so that this event is responsible for the TXNIP induction and NLRP3 inflammasome formation. In this regard, Li et al. [84] demonstrated that salicylate and AICAR, through the activation of AMPK, inhibited ROS production and the subsequent recruitment of the dynamin-related protein 1 (Drp1) on the mitochondrial membrane preventing mitochondrial fission and ER stress, thus, linking mitochondrial dysfunction to ER stress and OS in the generation of endothelial disturbances. Previously, Dong et al. [80] demonstrated that the AMPK activation by AICAR, metformin, and simvastatin suppresses ER stress through the inhibition of NOX-derived ROS and SERCA oxidation in glycated and oxidized-LDL- (HOG-LDL-) induced ED. Metformin, in particular, is widely used in diabetic patients and has been shown to be a strong activator of AMPK in vasculature [86–89]. AMPK activation following metformin administration had a therapeutic effect on HFD-fed mice with the inhibition of ER stress and OS and the restoration of EDD and NO production [67]. These effects were mediated by the interaction with the proliferator-activated receptor *δ* (PPAR*δ*) that is responsible for the upregulation of important pathways involved in lipid metabolism [67, 90]. Similarly, a recent work Choy et al. demonstrated that paeonol exerted a protective effect against tunicamycin-induced ER stress and the subsequent ED via activation of the AMPK/PPAR*δ* signalling pathway [91]. AMPK activation and its beneficial effects on endothelium functions are also involved in the molecular activity of mangiferin. The xanthonoid mangiferin was shown to be effective in high-glucose-induced ED by inhibiting ER stress and ER stress-dependent OS, and as for other AMPK activators, the inhibition of NLRP3 inflammasome allowed restoration of NO production and endothelial homeostasis [92]. Still concerning high-glucose-induced ED, cobalt (III) protoporphyrin IX chloride (CoPP) prevented ER stress, reduced inflammation and apoptosis, and improved endothelium functions and angiogenesis through the induction of NO release and vascular endothelial growth factor A (VEGFA) expression [93]. All these effects were mediated by CoPP-mediated induction of HO-1 [93]. A variety of other novel inhibitors of ER stress including fenofibrate, salidroside, and sodium hydrogen sulfide also have shown to be effective in the restoration of ER stress-dependent ED [94–96].

Another promising approach to reduce ER stress is represented by the upregulation of the ER folding capacity of ER chaperones or by the use of chemical chaperones. Tauroursodeoxycholate (TUDCA) and sodium phenylbutyrate (PBA) are two chemical chaperones previously approved by the Food and Drug Administration (FDA) for the treatment of, respectively, primary biliary cirrhosis and urea-cycle disorders and several diseases associated to ER stress and OS [97–100]. Interestingly, TUDCA and PBA have also displayed cardioprotection effects and therapeutic function on some CVDs such as ischemia/reperfusion and atherosclerosis [101–103]. Regarding the potential use of TUDCA and PBA for the treatment of ED, Walsh et al. demonstrated that oral administration of TUDCA reduced hyperglycemia-induced ED in humans [104]. In addition, the extensive use of TUDCA and PBA as chemical inhibitors of ER stress revealed their ability to inhibit ER stress-dependent features of ED such as EDD reduction, reduced eNOS phosphorylation, inflammatory response, and ROS production in experimental models of ED including hypertension [70, 78, 105], hyperglycemia [106–108], hyperhomocysteinemia [77], and hyperlipidemia [83, 84].

Another therapeutic strategy to neutralise ER stress-induced ED is the modulation of Bip/GRP78, PDI or Ero1 activity. In particular, a screening study, aimed at the discovery of Bip/GRP78 inducers, identified the compound BIX (Bip inducer X). BIX was found to induce Bip/GRP78 expression via the ATF6 pathway and to have protective effects towards ER stress-dependent apoptosis of neuroblastoma cells [109]. More interestingly, BIX intracerebral administration in ischemic mice reduced the area of infarction suggesting its potential use also in an ischemic heart [109].

Another promising, therapeutic approach is the targeting of Ero1. With this regard, Blais et al. identified the small Ero1*α* inhibitor EN460, reporting that this molecule interacted specifically with the active form of Ero1*α* and prevented its reoxidation [110]. In the same work, the authors found that the continuous exposure to a low concentration of EN460 protected the ER stress-sensitive PERK^−^/^−^ mouse embryonic fibroblasts from the exposure to tunicamycin, suggesting the potential use of Ero1*α* inhibitors in the protection against the consequences of severe ER stress in mammalian cells.

Similarly, in the same year, Pal et al. demonstrated that curcumin and masoprocol preserved PDI from S-nitrosylation during cycles of OS, protecting its functional integrity [111]. In particular, curcumin is a recognised anti-inflammatory and antioxidant drug, whose beneficial effect is well known for several diseases including cancer, diabetes, neurological, and CVDs thanks to its capacity to augment the activity of different antioxidant enzymes other than PDI [112, 113]. Only recently, curcumin was found to inhibit ER stress, to reduce insulin resistance through the inhibition of the JNK/insulin receptor substrate-1 (IRS-1) signalling, and to promote autophagy in endothelial cells exposed to palmitate, thus emphasizing its possible therapeutic outcome in ED [114].

An alternative strategy for mitigating ER stress is the modulation of individual UPR pathways such as PERK/eukaryotic initiation factor 2*α* (eIF2*α*) and IRE1/XBP1. These compounds revealed potential therapeutic features in several diseases related to ER stress including neurodegenerative and metabolic disorders, cancer, inflammatory disorders, and finally CVDs [115, 116].

With regard to the modulators of the PERK/eIF2*α* axis, several small molecules have been identified. This class includes salubrinal, a small compound that prevents the dephosphorylation of eIF2*α* through the inhibition of GADD34 and CReP, the two enzymes that direct the activity of the eIF2*α* protein phosphatase PP1 [117]. Salubrinal showed powerful protection from ER stress in several conditions [117–119] including myocardial infarction [120, 121] and oxidized-LDL-mediated ED [122]. On the contrary, recent studies found that salubrinal could potentiate lipid-induced ER stress with cytotoxic outcome [123, 124] suggesting that salubrinal employment in CVDs has to be accurately evaluated in clinical conditions.

Similarly to salubrinal, guanabenz, which is FDA-approved for the treatment of hypertension, increases eIF2*α* phosphorylation during ER stress condition through the inhibition of the CReP/PP1 complex [125].

Among the molecules that act directly on the PERK protein, GSK2606414 and GSK2656157 inhibit PERK phosphorylation showing promising anticancer activity [126, 127] and reduced development of prion disease in prion-infected mice [128]. Recently, ex vivo treatment of mouse mesenteric arteries with GSK2606414 was found to counteract the positive effect on vascular function and eNOS phosphorylation deriving from the overexpression of a longevity-associated genetic variant of the bactericidal/permeability increasing fold-containing-family-B-member-4 (LAV-BPIFB4) [129]. This work suggests that the potential therapeutic use of GSK2606414 in CVD could be negated in patients carrying the LAV-BPIFB4 genetic variant. In addition, or as an alternative, to the modulation of PERK/eIF2*α* signalling, the inhibition of the IRE1/XBP1 pathway can also be achieved to impair UPR in ER stress-dependent diseases. IRE1/XBP1 signalling can be impaired by inhibiting either IRE1 kinase activity or IRE1 RNAse activity. STF-083010, 4*μ*8C, MKC-3946, toyocamycin, and salicylaldehydes are small molecules targeting IRE1*α* RNAse activity and blocking XBP1 mRNA splicing and regulated IRE1-dependent decay of mRNA (RIDD) [130–134]. In contrast, APY29 or sunitinib inhibited IRE1*α* kinase activity without affecting oligomerization and RNAse activity while both activities were impaired by compound 3 [135, 136].

Overall, the efficacy of these molecules has been tested in vitro and in few in vivo models of various diseases, and no data are available from models of CVD. However, given their therapeutic potential, it will be interesting to investigate their clinical and biological effects on animal and cellular models of ER stress-dependent ED and CVD.

## 6. Conclusive Remarks

CVDs represent the most common cause of death worldwide, and although the clinical management and the prevention strategies have improved remarkably, they are still a public health issue in developed countries. Therefore, the discovery of new targets for the development of innovative therapeutic approaches for CVDs remains a fundamental mission of medical science, also considering that in the future this matter will be even more critical in view of the rise in life-expectancy levels in the population.

In this review, we extensively discussed the connections between ER stress, UPR, and OS in the pathogenesis of CVDs derived from ED. Although many aspects are only in part clear, for example, the contribution of each of the three branches of UPR and how it changes in acute and chronic ED, the ER stress and its signalling response certainly represent a promising system to design new molecules and elaborate new therapeutic methodologies for the management of ED. In this context, we examined how the signalling pathways of the UPR could be modulated to establish therapeutic strategies to alleviate ED. Such a result has been achieved either by enhancing the antioxidative mechanisms or by inhibiting prooxidative properties of the UPR pathways. The choice between the two strategies depends on the different temporal outcomes of the adaptive response with regard to the prooxidative and proapoptotic response, the first being activated earlier and the second upon prolonged stress induction.

Another factor that should be taken into account might be the effect of UPR inhibition on other tissues not experiencing ER stress. For example, PERK expression is essential for pancreatic *β* cells, while IRE1*α* RIDD activity is expressed in basal conditions and is essential to maintain ER homeostasis [20, 137]. Moreover, unexpected effects could come by the inhibition of UPR transducers also in the targeted tissue. For example, the RIDD activity of IRE1 is crucial for the regulation of microRNA expression during UPR activation [138, 139]; therefore, inhibition of IRE1 RIDD activity could have deleterious effects on the expression of the microRNA targets. The conflicting data regarding UPR inhibition (such as those concerning salubrinal, as reported previously) reveal the complexity of UPR response and indicate that its modulation may exert both protective and toxic effects depending on the nature of the insult. These considerations highlight that future efforts are necessary to solve this puzzle in order to develop new clinical protocols for the management of ED.

Therefore, further studies are needed in order to define the optimal targets for each specific clinical condition, develop novel drugs, and prevent possible side effects deriving from the UPR perturbations.

## Abbreviations

AICAR: Aminoimidazole carboxamide riboside
AMPK: AMP-activated protein kinase
ASK1: Apoptosis signal-regulating kinase 1
ATF4: Activating transcription factor 4
ATF6: Activating transcription factor 6
Bip/GRP78: Binding immunoglobulin protein/78 kDa glucose-regulated protein
BIX: Bip inducer X
BPIFB4: Bactericidal/permeability increasing fold-containing-family-B-member-4
CaMKII: Ca^2+^/calmodulin-dependent protein kinase II
CHOP: C/EBP homologous protein
CoPP: Cobalt (III) protoporphyrin IX chloride
COX-2: Cyclooxygenase-2
CReP: Constitutive reverter of eIF2*α* phosphorylation
CVDs: Cardiovascular diseases
Drp1: Dynamin-related protein 1
ED: Endothelial dysfunction
EDD: Endothelium-dependent dilatation
eIF2*α*: Eukaryotic initiation factor 2*α*
eNOS: Endothelial NOS
ER: Endoplasmic reticulum
ERAD: ER-associated degradation
Ero1: ER oxidoreductase 1
ERQC: ER quality control
ETC: Electron transport chain
FDA: Food and drug administration
GADD34: Growth arrest and DNA damage-inducible protein
GSH: Glutathione
HBP: Hexosamine biosynthetic pathway
HC: Homocysteine
HFD: High-fat diet
HO-1: Heme oxygenase 1
HOG-LDL: Glycated and oxidized-LDL
IP3R: Inositol-1,4,5-trisphosphate receptor
IRE1: Inositol-requiring kinase 1
IRS-1: Insulin receptor substrate-1
ISR: Integrated stress response
JNK: c-Jun N-terminal kinase
KEAP1: Kelch-like ECH-associated protein 1
LAV-BPIFB4: Longevity-associated variant of BPIFB4
MAM: Mitochondrial-associated ER membranes
NLRP3: NOD-like receptor family pyrin domain containing 3
NO: Nitric oxide
NOS: Nitric oxide synthase
NOX: NADPH oxidase
NRF2: Nuclear factor erythroid 2-related factor 2
OS: Oxidative stress
PBA: Sodium phenylbutyrate
PDI: Protein disulfide isomerase
PDIA5: Protein disulfide isomerase A5
PDIA6: Protein disulfide isomerase A6
PERK: Pancreatic ER kinase
PKR: Protein kinase R
PP1: Protein phosphatase 1
PPAR*δ*: Proliferator-activated receptor *δ*
RIDD: Regulated IRE1-dependent decay of mRNA
ROS: Reactive oxygen species
RyR: Ryanodine receptor
S1P: Site 1 protease
S2P: Site 2 protease
SERCA: Sarcoplasmic reticulum calcium transport ATPase
TDAG51: T cell death-associated gene 51
TRAF2: Tumor necrosis factor *α*-associated receptor 2
TRX: Thioredoxin
TUDCA: Tauroursodeoxycholate
TXNIP: Thioredoxin-interacting protein
UDP-GlcNAc: UDP-N-acetylglucosamine
UPR: Unfolded protein response
VEGFA: Vascular endothelial growth factor A
XBP1: X-box-binding protein 1.
